# Revisit of the Association between Cytomegalovirus Infection and Invasive Fungal Infection after Allogeneic Hematopoietic Stem Cell Transplantation: A Real-World Analysis from a High CMV Seroprevalence Area

**DOI:** 10.3390/jof8040408

**Published:** 2022-04-16

**Authors:** Tsung-Jang Yeh, Ching-I Yang, Chien-Tzu Huang, Min-Hung Wang, Tzer-Ming Chuang, Ya-Lun Ke, Yuh-Ching Gau, Jeng-Shiun Du, Hui-Ching Wang, Shih-Feng Cho, Ching-Ping Lee, Chin-Mu Hsu, Hui-Hua Hsiao, Yi-Chang Liu

**Affiliations:** 1 Division of Hematology & Oncology, Department of Internal Medicine, Kaohsiung Medical University Hospital, Kaohsiung Medical University, Kaohsiung 807, Taiwan; aw7719@gmail.com (T.-J.Y.); febeey0118@gmail.com (C.-I.Y.); niamycat@gmail.com (C.-T.H.); dhlsy01128@gmail.com (M.-H.W.); benjer6@gmail.com (T.-M.C.); a9601082@gmail.com (Y.-L.K.); cheesecaketwin@gmail.com (Y.-C.G.); ashiun@gmail.com (J.-S.D.); joellewang66@gmail.com (H.-C.W.); sifong96@gmail.com (S.-F.C.); ping890218@gmail.com (C.-P.L.); e12013@gmail.com (C.-M.H.); huhuhs@kmu.edu.tw (H.-H.H.); 2 Graduate Institute of Clinical Medicine, College of Medicine, Kaohsiung Medical University, Kaohsiung 807, Taiwan; 3 Specialist Nurse and Surgical Nurse Practitioner Office, Kaohsiung Medical University Hospital, Kaohsiung Medical University, Kaohsiung 807, Taiwan; 4 Faculty of Medicine, College of Medicine, Kaohsiung Medical University, Kaohsiung 807, Taiwan; 5 Cell Therapy and Research Center, Kaohsiung Medical University Hospital, Kaohsiung 807, Taiwan

**Keywords:** allogeneic hematopoietic stem cell transplantation, cytomegalovirus infection, invasive fungal infection

## Abstract

Infection is a major complication after allogeneic hematopoietic stem cell transplantation (allo-HSCT) especially cytomegalovirus (CMV) infection and invasive fungal infection (IFI). Taiwan is a high CMV seroprevalence area. Our study aimed to evaluate the incidence, risk factors, the impact on survival of CMV infection (including reactivation and disease) and the association of CMV infection and IFI in recipients after allo-HSCT during the first 100 days after transplantation. This was a retrospective study including 180 recipients of allo-HSCT. A total of 99 patients had CMV reactivation, and nine patients had CMV diseases. There were more mismatched donors, more ATG usage and more transplantation from CMV IgG-negative donor in patients with CMV reactivation. There was no survival difference in patients with or without CMV reactivation. A total of 34 patients had IFIs, and IFI after allo-HSCT was associated with significantly inferior survival. Patients with CMV reactivation did not increase the incidence of overall IFI, but they did result in more late-onset (>40 days) IFI (*p* = 0.056). In this study, we demonstrated real-world data of CMV infection and IFI from a high CMV seroprevalence area.

## 1. Introduction

Allogeneic hematopoietic stem-cell transplantation (allo-HSCT) is an effective and intensive treatment option to cure many hematologic diseases, especially acute leukemia. Disease control is the main purpose of allo-HSCT; however, the management of complications, especially infection after transplant, is unneglectable.

From the Center for International Blood and Marrow Transplant Research (CIBMTR) database, infection accounts for 16% and 22% of the reason of mortality within 100 days post-transplant in matched related HSCT and matched unrelated HSCT, respectively [1,2]. Infection remained one of the leading causes of morbidity and mortality after allo-HSCT, and cytomegalovirus (CMV) infection and invasive fungal infection (IFI) are two important topics that cannot be ignored. After allo-HSCT, the incidence of CMV reactivation was reported to be 30% to 70% [3], and IFIs ranged from 7% to 32% from prior studies [4,5,6,7,8,9,10,11,12,13,14].

CMV seroprevalence is highest in South America, Africa, and Asia but it is relatively lower in Western Europe and the United States from previous review [15]. In Taiwan, the CMV prevalence is very high, for more than 90% from local studies, and it leads to the importance of CMV infection in the Taiwanese population [16,17]. CMV infection usually appears within the first 100 days after allo-HSCT and affects mainly the lungs and the gastrointestinal tract [18]. In addition to the direct impact of CMV end-organ disease, CMV is also associated with an increased incidence of opportunistic infections and graft-versus-host disease (GVHD) in allo-HSCT recipients [19,20]. Several factors had been documented to be associated with CMV reactivation, such as CMV serostatus of the donor and recipient, the intensity of conditioning regimen, the use of total body irradiation, fludarabine-containing regimens, the use of T-cell-depleting agents (for example, antithymocyte globulin and alemtuzumab), the use of high-dose corticosteroids, and the presence of acute or chronic GVHD [21,22].

Risk factors for IFI were similar to CMV infection, such as extended use of immunosuppressive drugs, conditioning-related prolonged neutropenia and lymphopenia, corticosteroid use, acute or chronic GVHD, and rejection [23]. Some more specific factors had also been reported, such as antiviral treatment-related immunosuppression, CMV-related immunosuppressive modulation of the host immune system, iron overload, and genetic predisposition [23,24,25,26,27,28]. What is more, the timing of early (≤40 days after HSCT) and late (41–100 days after HSCT) IFIs also matters. Prior studies had demonstrated age, prior history of IFI, HLA mismatch, long-time neutropenia, and acute GVHD as risk factors for early IFI; prior history of IFI, corticosteroid therapy, CMV disease, and chronic GVHD are risk factors for late IFI [14,28]. In addition, Candida was the most common pathogen for early IFI, and mold was the most frequent organism for late IFI [14].

To date, several studies have tried to prove the association between CMV infection and IFIs, but the results were inconsistent [14,23,28,29,30,31,32,33,34]. Most of the studies were from early research, and the definitions of CMV infection (CMV reactivation or CMV disease) and IFI (early or late IFI) were not identical. Therefore, based on the importance of CMV infection and IFI after allo-HSCT, we reported a real-world analysis of CMV infection and IFI in adult patients undergoing allo-HSCT in a Taiwanese medical center.

## 2. Materials and Methods

### 2.1. Study Design and Patient Selection

A total of 180 adult patients undergoing allo-HSCT from July 2008 to June 2019 at Kaohsiung Medical University Hospital were retrospectively reviewed. Peripheral blood was the only source of hematopoietic stem cell. The data contained detailed information on clinical characteristics, age, gender, underlying diseases, disease status at transplantation, donor type, conditioning regimens (myeloablative or reduced intensity regimens), the use of total body irradiation (TBI), the use of anti-thymocyte globulin (ATG), acute GVHD, and CMV serostatus of the recipient and donor. CMV serostatus was recorded by the immunoglobin M (IgM) and immunoglobin G (IgG) against CMV before allo-HSCT. Every patient had at least followed for 180 days after allo-HSCT or until any event had occurred. This study was approved by the Institutional Review Board and Ethics Committee of Kaohsiung Medical University Hospital (KMUHIRB-E(I)-20200361).

### 2.2. Conditioning Regimen

Myeloablative conditioning regimens included BuCy2 (busulfan: 3 mg/kg per day from day −7 to day −4; cyclophosphamide: 60 mg/kg per day, from day −3 to day −2) or TBI (1200 cGy/6 fractions from day −6 to day −4) plus cyclophosphamide (60 mg/kg per day, from day −3 to day −2). The reduced-intensity conditioning regimen included TBI (200 cGy, day −7), fludarabine (30 mg/m^2^ per day, from day −6 to day −2), and cyclophosphamide (60 mg/kg per day, from day −3 to day −2) or fludarabine (30 mg/m^2^ per day, from day −6 to day −2) with busulfan (2.4 mg/kg per day, from day −5 to day −2). As for the regimen of haploidentical HSCT, we used FB2 (cyclophosphamide 14.5 mg/kg/day for 2 days, fludarabine 30 mg/m^2^/day for 5 days and busulfan 3.2 mg/kg/day for 2 days) or FB4 (cyclophosphamide 14.5 mg/kg/day for 2 days, fludarabine 30 mg/m^2^/day for 5 days and busulfan 3.2 mg/kg/day for 4 days) with standard post-transplantation cyclophosphamide (PTCy) protocol.

### 2.3. Graft-Versus-Host Disease Prophylaxis

In this study, cyclosporine (1.5 mg/kg twice a day since day −1 and then adjusted according to trough level) and short-course methotrexate (15 mg/m^2^ on day 1 and 10 mg/m^2^ on days 3, 6 and 11) were the major immunosuppressant. A serum cyclosporine trough level of 150–250 ng/mL was the targeted concentration. The cyclosporine dose was generally tapered after day 90. Myfortic acid was used since day −2 at a dose of 720 mg twice daily and was generally discontinued on day 60. For those patients undergoing haploidentical HSCT, post-transplant cyclophosphamide (PTCy) was performed.

Rabbit anti-thymocyte globulin (ATG) was routinely given to patients without matched sibling donors at 2 mg/kg/day from day −4 to day −2. After transplantation, acute and chronic GVHD was evaluated and graded according to standard criteria. If grade II-IV acute GVHD was presented, steroid was used and adjusted according to clinical condition.

### 2.4. CMV Monitoring

All recipients were monitored with CMV PCR weekly until day 100. The peripheral blood leukocyte DNA was extracted by GFX genomic blood DNA purification kit (Amersham Biosciences, Piscataway, NJ, USA), and CMV DNA was detected by PCR using primers and conditions as previously described [30]. The CMV PCR was qualitative before and became quantitative since 2016. The CMV pp65 antigenemia assay was examined simultaneously to March 2019. The CMV antigenemia assay was performed using the commercially available kit (CINAkit, Argene, Varihes, France), which allowed the indirect immunofluorescence detection of lower matrix protein pp65 of CMV in peripheral blood leukocytes. A single stained cell seen per slide indicated a positive antigenemia, with a sensitivity estimated to be one positive cell per 1 × 10^5^ cells.

All recipients received acyclovir as viral prophylaxis form day −7 to day −1, and no CMV-specific prophylaxis was given in this study. There is no routine use of viral prophylaxis in the post-transplantation period.

### 2.5. CMV Infection, CMV Disease, and Pre-Emptive Therapy

The definition of CMV infection and CMV disease was based on the criteria reported previously [35]. The cut-off value of 1000 copies/mL by quantitative CMV PCR test was used to define CMV infection. Before 2019, the presence of CMV pp65 antigenemia or two consecutive positive results of qualitative CMV PCR was defined as CMV infection. When CMV infection was first documented, pre-emptive therapy with ganciclovir was initiated. Ganciclovir was administered at a dose of 5 mg/kg twice daily for 14 days followed by 5 mg/kg once daily. The treatment was stopped when two consecutive negative results on CMV PCR or CMV pp65 antigenemia were obtained. In cases of CMV disease, at least 21 days of ganciclovir was prescribed. CMV immunoglobin (500 mg/kg every other day for 20 days) was also added according to the severity of CMV disease.

### 2.6. Diagnostic Strategy of Invasive Fungal Infection

In patients with febrile neutropenia (temperature >38 °C recorded twice or >38.5 °C recorded once), a baseline diagnostic work-up was performed including blood cultures, microbiological and radiological examinations, and followed by empirical antibacterial therapy. If a patient had persisting fever after 4 days of antibacterial therapy or a patient had fever relapsing after 48 h of defervescence, invasive fungal infection was suspected, and an intensive diagnostic work-up was underwent.

Due to the high prevalence of pulmonary fungal infection in patients after allo-HSCT, we evaluated patients who suffering from pneumonia with chest radiographs and sometimes high-resolution chest CT scan. Any well-circumscribed dense lesions, air crescent sign, cavity, wedge-shaped, and segmental or lobar consolidation from chest radiographs or CT scan met the criteria of clinical features and were regarded as the evidence of pulmonary IFI. Bronchoscopy with bronchoalveolar lavage (BAL) was performed if clinically appropriate in order to obtain proper specimen for culture and mycologic tests. If sino-nasal diseases were suspected, ENT consultation was performed for careful examinations and local treatment, such as biopsy.

Testing for values of galactomannan (GM) in serum or BAL fluid had used as a diagnostic test for invasive aspergillosis in our hospital. The cut-off values of serum and BAL fluid GM tests were 0.5 and 1.0, respectively.

IFIs were defined and graded according to the 2020 Revision and Update of the Consensus Definitions of Invasive Fungal Disease from the European Organization for Research and Treatment of Cancer and the Mycoses Study Group Education and Research Consortium (EORTC/MSGERC) [36]. We classified patients into proven, probable and possible IFIs based on host factors, clinical features and mycological evidence. Proven diagnosis demanded histopathological findings or positive culture from a primary sterile site. Probable IFI required the presence of a host factor, a clinical feature and mycologic evidence. Cases that met the criteria for a host factor and a clinical feature but for which mycological evidence had not been found were considered possible IFI [36]. All patients in our study met the criteria of host factors; we focused on the clinical features and mycologic evidence to classify our patients. If patients met the criteria of proven, probable or possible IFIs, IFIs were confirmed, and proper antifungal treatment were started. Overall, we analyzed the IFI episodes during first 100 days post-transplantation and further classified into early-onset IFIs (≤40 days) and late-onset IFIs (>40 days).

### 2.7. Statistical Analysis

The following variables were analyzed to determine the risk factors for CMV infection and CMV disease, including age, gender, underlying diseases, disease status at transplantation, donor type, CMV serostatus of the recipient and donor, conditioning regimens, acute GVHD, and early or late IFIs. Only the first episode of CMV infection was investigated in this study. Overall survival was defined from diagnosis to death. The Kaplan–Meier survival analysis was used to calculate the survival and the cumulative incidence of CMV infection, and the difference between each of the two survival curves was estimated using the log-rank test. Multivariate Cox proportional hazards regression analysis was performed to identify prognostic factors for CMV infection and disease. The statistical significance level of the *p*-value was set at 0.05. All statistics were calculated using SPSS 20.0 software (SPSS Inc., Chicago, IL, USA).

## 3. Results

### 3.1. Baseline Characteristics of Patients

A total of 180 allogeneic HSCT recipients were included. Median age at allo-HSCT was 39.22 (±11.76) years, and 91 (50.6%) patients were male. More than half of the patients were diagnosed with acute leukemia (47.2% patients with acute myeloid leukemia and 26.7% patients with acute lymphoblastic leukemia). Other diseases included chronic myeloid leukemia, severe aplastic anemia, myelodysplastic syndrome, and non-Hodgkin lymphoma. There were 50.6% patients who underwent allogeneic HSCT in a remission status (first complete remission plus second complete remission) and 41.4% patients without remission. For the donor origin, 50% patients had HLA full-matched sibling donor (MSD), and 23.3% had HLA full-matched unrelated donor (MUD). Mismatched donors, including mismatched sibling donor, mismatched unrelated donor and haploidentical donor, was 26.7%. For the intensity of conditioning regimen, 94.4% of patients received myeloablative regimen, including 70.6% that were TBI-based. ATG was used in 49.4% of patients. CMV IgG was positive in 92.2% (166 of 180) of the recipients and 80.6% (145 of 180) of the donors, suggesting a high seroprevalence rate in our cohort. Paired donor/recipient serology status showed that 139 (77.2%) were D+/R+, 27 (15%) were D−/R+, 6 (3.3%) were D+/R−, and 8 (4.4%) were D−/R−. All patients were engrafted successfully after allogeneic HSCT, and the median time of WBC engraftment was 12.26 days. The clinical characteristics are listed in Table 1.

### 3.2. CMV Reactivation and CMV Disease

The overall incidence of CMV reactivation within the first 100 days after HSCT is 55.0% (99 of 180). The median time of first CMV reactivation was 27.58 days. A total of nine patients developed CMV disease (5.0%) at a median of 39.56 days. CMV pneumonia was diagnosed in three patients, CMV enterocolitis in four patients, and CMV retinitis in two patients (Table 2). Tissue sampling for pathology review was performed in all patients with CMV disease, including bronchoalveolar lavage for CMV pneumonia and endoscopic biopsy for CMV enterocolitis. CMV retinitis was diagnosed by experienced ophthalmologist with fundus examination, including one patient with documented CMV DNA in vitreous fluid. Patients with CMV disease were all treated with intravenous ganciclovir initially. CMV immune globulin was also added as adjunctive treatment. Among these patients, seven patients died, but only one of these deaths can be directly attributed to CMV disease. The causes of death in six other patients included two with disease relapse, one with severe GVHD and three with bacterial pneumonia with documented pathogens. The attributable mortality rate of CMV disease in our cohort was 0.6% (1 of 180).

### 3.3. Comparisons between Patients with and without CMV Reactivation

The median age of patients with CMV viremia was similar to patients without CMV viremia (39.14 vs. 39.31 years). Donor origin was different between two groups (*p* = 0.014). There were more matched sibling donors in the group without CMV viremia and more matched unrelated donor and mismatched donors in the group with CMV viremia. In addition, more patients received ATG as GVHD prophylaxis in the CMV viremia group (*p* = 0.007). Donor/recipient CMV serology status was also statistically different between groups (*p* = 0.019). There was more donor negative and recipient positive (D−/R+) in patients with CMV viremia. CMV viremia was seen in 54.0% (75 of 139) of D+/R+ patients, in 74% (20 of 27) of D−/R+ patients, in 50% (3 of 6) of D+/R− patients, and 12.5% (1 of 8) of D−/R− patients. There were no statistical differences between the two groups with regard to age, gender, underlying disease, disease status at transplantation, the intensity of conditioning regimen and the degree of acute GVHD.

### 3.4. Risk Factors for CMV Reactivation

Several factors had been documented to be associated with CMV reactivation in published articles, such as CMV serostatus of the donor and recipient, the intensity of conditioning regimen, the use of total body irradiation, the use of T-cell-depleting agents (for example, ATG), the use of high-dose corticosteroids and the severity of acute GVHD [21,22,37,38,39]. As a result, we chose age, gender, donor origin, the intensive of conditioning regimen, the use of total body irradiation, the use of ATG, the severity of acute GVHD and the CMV serostatus of donor and recipient for further analysis.

In univariate analysis, donor origin (matched sibling donor vs. matched unrelated or others) and the use of ATG were identified as risk factors for CMV reactivation (Table 3). The variance inflation factor (VIF) test indicated collinearity between donor origin and the use of ATG. Therefore, ATG was suggested to be removed from the multivariate model. After adjustment, donor origin still showed association to CMV viremia with statistical significance.

**Figure 1:**
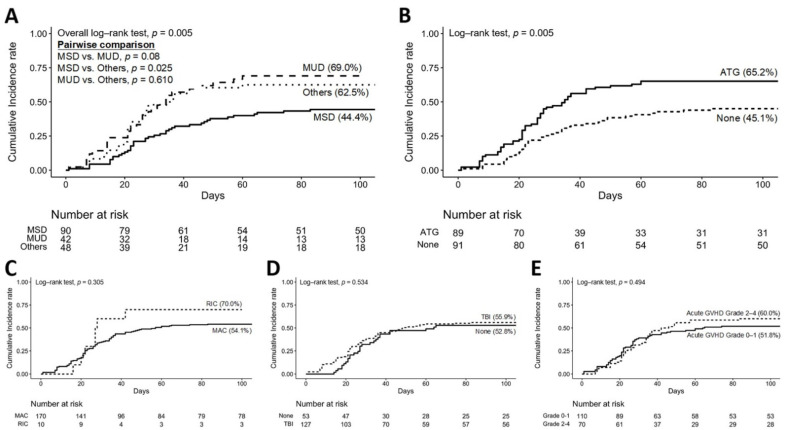
Cumulative incidence rate of CMV reactivation within 100 days after transplantation. (**A**) different donor origins, (**B**) the usage of ATG or not, (**C**) different intensity of conditioning regimen, (**D**) the usage of TBI or not, and (**E**) different acute GVHD grades.

The cumulative incidence of CMV viremia within the first 100 days was 44.4% in MSD, 62.5% in mismatch donors, and 69.0% in MUD (overall log-tank *p* = 0.005; Figure 1A). The cumulative incidence of CMV viremia was 65.2% in patients who received ATG and 45.1% in patients who did not receive ATG (log-tank *p* = 0.003, Figure 1B). There were no significant differences between the cumulative incidence of other factors, such as the intensity of conditioning regimen, the usage of TBI, and different acute GVHD grades (Figure 1C–E).

### 3.5. Impact of CMV Reactivation and CMV Disease on Survival

The overall survival for all patients after HSCT was 84.4% (152 of 180) at 100 days, 74.4% (134 of 180) at 180 days and 55.6% (100 of 180) at 1 year. Patients who developed CMV viremia showed no statistically significant difference in 180-day survival (log-rank *p* = 0.2175) and overall survival (log-rank *p* = 0.3234). Patients who developed CMV disease also showed no significant survival disadvantage before day 180 (log-rank *p* = 0.3493) and overall survival (log-rank *p* = 0.2783).

### 3.6. The Association of CMV Reactivation and Invasive Fungal Infection

The overall incidence of invasive fungal infection during the first 100 days after allo-HSCT was 18.9% (34 of 180). Median diagnosis time of IFI were 39.88 days. Seven patients had proven IFIs, one patient had probable IFI, and 26 patients had possible IFIs according to EORTC/MSGERC 2020 criteria [36]. The characteristics of CMV reactivation and IFIs are shown in Table 4.

The incidence of IFI (proven, probable and possible) was higher in patients with CMV reactivation compared with those without (20.2% vs. 17.3%) but there was no statistically significant difference (*p* = 0.381).

Based on previous studies, CMV infection was regarded as a risk factor of late-onset (>40 days) IFI rather than early-onset (≤40 days) IFI [14,28,30,40]. Among the 34 IFI patients in our cohort, 20 patients had early-onset IFIs and 14 patients had late-onset IFIs. There was no statistically significant difference between early-onset IFIs in patient with and without CMV reactivation (9.1% vs. 13.6%, *p* = 0.237). However, there was a trend toward more patients developing late-onset IFI in patient with CMV viremia (11.1% vs. 3.7%, *p* = 0.056) (Table 4).

### 3.7. Impact of Invasive Fungal Infection and CMV Reactivation on Survival

**Figure 2:**
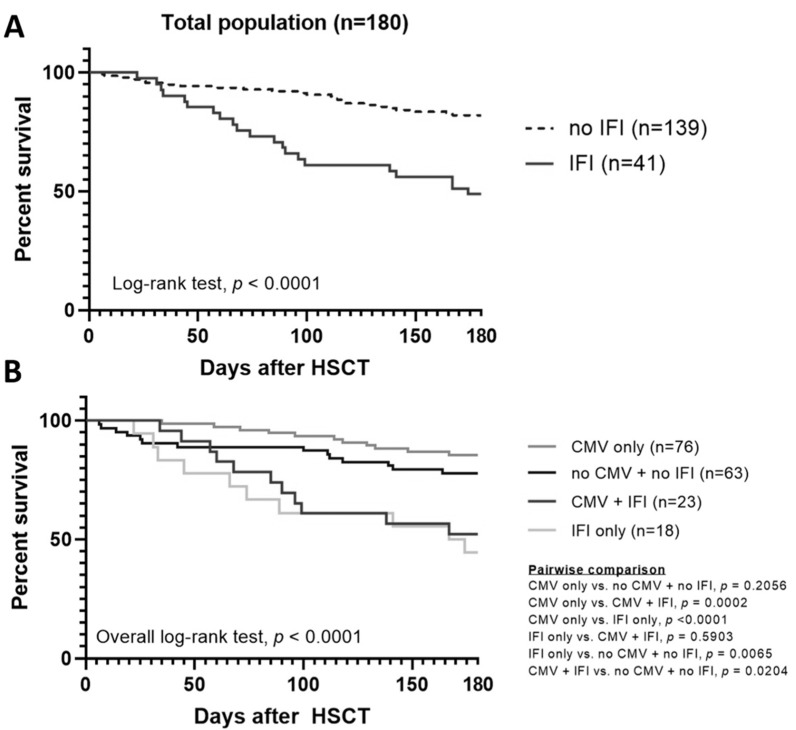
Kaplan–Meier 180-day survival estimates after HSCT. (**A**) IFI vs no IFI, and (**B**) four subgroups according to CMV reactivation and IFI.

In our cohort, patients who developed IFI had a lower survival rate than patients who did not have IFI (log-rank *p* < 0.0001, Figure 2A). In the 99 patients who had CMV reactivation after allo-HSCT, 76 patients further developed IFI and 23 patients did not. In those who had CMV reactivation and IFI, the mortality rate was 47.8% (11 of 23); in those who had CMV reactivation but no IFI, the mortality rate was 14.5% (11 of 76). There was a statistically significant difference (log-rank *p* = 0.0002).

We further separated patients into four subgroups according to CMV reactivation and IFI: group (A) patients had CMV reactivation and IFI, group (B) patients had CMV reactivation without IFI, group (C) patients did not have CMV reactivation but had IFI, and group (D) patients did not have either CMV reactivation or IFI. The overall survival of these four groups is shown in Figure 2B (overall log-rank *p* < 0.0001). Patients who developed IFI with or without CMV viremia (group A and group C) had a significantly lower survival rate than other groups (group B and group D), and there was no survival difference between patients with both CMV reactivation and IFI (group A) and patients with IFI only (group C) (*p* = 0.5903). For those patients who did not develop IFI (group B and group D), there was also no survival difference (*p* = 0.2056).

## 4. Discussion

This study provides a large cohort with consecutive patients receiving allo-HSCT that contained detailed clinical longitudinal information, including monitoring protocols, treatment regiments and outcomes, which is not easily obtained in a population-based study. Clinical outcomes and survival analysis reflect response to real-world interventions, which may not be similar with results of clinical trials.

Infection rates are strongly associated with local epidemiological features. An observation of serial changes of the rate and outcomes of CMV reactivation following allo-HSCT is important in a high CMV seroprevalence area. Compared with prior early studies in Taiwan, Liu et al. reported that the incidence of CMV reactivation was 45.3% in 117 allo-HSCT patients, and the survival rate was 56.7%. The incidence of CMV disease was 6.8% (8 of 117), and the mortality rate was 1.7% (2 of 117). Multivariate analysis showed that grade II–IV acute GVHD and ATG-containing conditioning regimen were associated with an increased risk of CMV reactivation. There was no survival disadvantage in patients who developed CMV reactivation or CMV disease [37]. Lin et al. reported an overall incidence of CMV reactivation of 39.0% in 82 allo-HSCT patients. Multivariate analysis revealed that ≥grade 3 acute GVHD was associated with CMV reactivation. There was a trend toward inferior overall survival in patients with CMV reactivation, but there was no statistical difference. They also noticed that patients with ≥grade 3 acute GVHD were more refractory to CMV treatment, so maybe CMV prophylaxis therapy should be considered in this group [38]. Later, Lin et al. reported an overall incidence of CMV reactivation of 55.0% in 80 allo-HSCT patients with acute leukemia. Cumulative incidence of CMV reactivation within 180 days was highest in patients who underwent haploidentical allo-HSCT (85.7%). Multivariate analysis revealed that haploidentical allo-HSCT and age were connected with increased CMV reactivation [39].

In this study, we analyzed 180 recipients of allo-HSCT. Factors associated with CMV reactivation were also collected. Baseline characteristics showed that donor origin (matched unrelated donor and mismatched donors), the use of ATG, and donor/recipient CMV serostatus (especially D−/R+) were statistically more in patients with CMV reactivation. Those results were compatible with previous studies [37,38,39]. The overall incidence of CMV reactivation within the first 100 days was 55.0% (99 of 180), and the incidence of CMV disease was 5.0% (9 of 180). Donor origin and the use of ATG are associated with CMV reactivation in univariant analysis. Due to the use of ATG being widely used in allo-HSCT with unrelated donors, the collinearity between donor origin and the use of ATG was very significant. However, even after we removed the use of ATG from multivariate analysis, donor origin was still associated with CMV reactivation with statistical significance. This result demonstrated that both factors, donor origin and the use of ATG, were both related to CMV reactivation. It proved again that ATG, a T-cell-depleting agent causing a delay in lymphocyte engraftment and prolonged time to absolute lymphocyte count recovery, is a risk factor for CMV reactivation as previous studies have shown [21,22,37].

Another common risk factor for CMV reactivation is the severity of acute GVHD. However, we could not demonstrate the significance in our cohort. Although the prevalence of CMV reactivation was more than 50% after allogeneic HSCT, it did not affect the survival of these patients. There was no disadvantage in survival in the first 180 days or overall survival in patients who developed CMV reactivation or CMV disease. It may contribute to early CMV detection (for example, weekly CMV antigen or PCR follow-up protocol) and prompt a pre-emptive strategy. Appropriate supportive care might also be essential in optimizing the outcome.

The incidence of CMV reactivation in this cohort (55%) was higher than the old study (45.3%) in 2012 [37]. We can attribute it to two possible reasons. First is the change of CMV virus detection method. We previously used only CMV pp65 antigenemia assay and then changed to CMV real-time PCR assay. However, CMV pp65 antigenemia assay was strongly influenced by neutropenia, which was very common in the post-HSCT period. Therefore, the detection of CMV viremia via real-time PCR in neutropenic patients is more suitable and may result in a better detection rate. The second reason was the change of CMV serostatus of donors. The prevalence of CMV seropositivity of the recipients was quite similar between this cohort and previous study (96.4% vs. 92.2%), but the CMV seropositivity of the donor was much different (92.9% vs. 80.6%) [37]. More CMV seronegative donors were noticed in this cohort. Although there are no recent data about the change of CMV epidemiology in Taiwan, we assume that the change of serology might be explained by the development of public health in recent decades. The risk of young people to be infected by CMV in daily lives decreased, so the prevalence of CMV seropositivity became lower. Therefore, the phenomenon resulted in more D–/R+ transplantation and increased the risk of CMV reactivation after HSCT.

Whether CMV reactivation as defined by PCR is a risk for IFIs is still to be determined, and it remains unknown for early or late IFIs. Several previous studies (often retrospective) did demonstrate CMV infection, either CMV reactivation or CMV disease, is a risk factor for IFI [14,23,28,29,30,31,32,33,34], and CMV disease was shown to confer a 4–7 times increased risk for IFI [28,29,30]. A meta-analysis about the association of CMV infection and IFI in allo-HSCT recipients demonstrated that CMV infection significantly increased the risk of IFIs with a pooled hazard ratio of 2.58. Post-transplant CMV infection and high-risk CMV serostatus (D–/R+) increased the risk of IFIs, but low-risk CMV serostatus (D–/R–) decreased the risk of IFIs [40]. However, most of the studies were published before 2010 and only focused on CMV disease [28,32,33,34]. As the detection improved, the incidence of CMV disease decreased in recent years. Furthermore, studies associated with CMV infection and IFI, published after 2010, started to focus on CMV DNAemia/pp65 antigenemia. Although some studies still showed correlation between CMV reactivation and IFI [14,29,30], there are some studies showing negative correlation [12,41,42]. In our study, we also found that there was no difference of IFI incidence between patients with and without CMV reactivation (20.2% vs. 17.3%). It might be due to the improvement of early detection of CMV reactivation in recent decades by either antigenemia assay or real-time PCR assay.

In this study, the incidence of IFI was 18.9% in the first 100 days after allo-HSCT. Unlike CMV reactivation, we noticed that patients who developed IFI had significantly inferior survival compared to patients did not have IFI.

We compared our cohort with prior smaller studies investigating IFIs following allo-HSCT in Taiwan. Liu et al. reported that the incidence of probable and proven IFI incidence was 7.4% of 31 allo-HSCT patients. Risk factors for IFI included EBMT risk score >2, prior history of IFI, type 2 diabetes mellitus, acute GVHD, extensive chronic GVHD, the development of post-transplant lymphoproliferative disorder, and the use of high-dose steroid. However, CMV infection, did not increase IFI (*p* = 0.907) [12]. Chien et al. evaluated invasive mold infections (IMIs) in 245 adult acute leukemia patients undergoing allo-HSCT. There were 6.9% patients who developed IMIs, and Aspergillus species were the most common pathogen. The significant risk factors predicting IMIs were unrelated donor transplantation, smoking, EBMT risk score >2, and moderate to severe chronic GVHD. CMV reactivation did not increase the risk of IFI, either (*p* = 0.297) [43].

In our cohort, we could not demonstrate the association between CMV reactivation and overall IFI just like these two studies in Taiwan [12,43]. However, if further evaluating the timing of IFI, especially in late-onset IFI, we did find a trend toward more patients developing late-onset IFI in patients with CMV reactivation just like other studies [14,28,30]. We also noticed that in patients with CMV reactivation, IFI resulted in an inferior 180-day survival and overall survival with statistical difference. In summary, regardless of CMV reactivation, patients with IFI had worse survival.

## 5. Conclusions

Infection complications after allo-HSCT, especially CMV infection and IFI, are a major factor of mortality. We demonstrated a real-world data for CMV infection and disease and studied the association with IFIs during the first 100 days after allo-HSCT from a high CMV seroprevalence area. Although CMV reactivation rate (55%) was high, it did not affect survival. Risk factors for CMV reactivation were donor origin, the CMV serostatus of donors and recipients, and the use of ATG. Unlike CMV reactivation, IFI after allo-HSCT resulted in shorter survival. There was a trend toward more patients developing late-onset IFI in the population with CMV reactivation, which warrants further investigation.

## Figures and Tables

**Table 1:** Baseline characteristics of patients receiving allo-HSCT.

Characteristics	Overall (*n* = 180)	No CMV Reactivation (*n* = 81)	CMV Reactivation (*n* = 99)	*p*
Age (years)	39.22 ± 11.76	39.31 ± 12.40	39.14 ± 11.27	
Age				0.810
<40	96 (53.3%)	44 (54.3%)	52 (52.5%)	
≥40	84 (46.7%)	37 (45.7%)	47 (47.5%)	
Gender				0.075
Female	89 (49.4%)	46 (56.8%)	43 (43.4%)	
Male	91 (50.6%)	35 (43.2%)	56 (56.6%)	
Diagnosis				0.145
AML	85 (47.2%)	32 (39.5%)	53 (53.5%)	
ALL	48 (26.7%)	26 (32.1%)	22 (22.2%)	
CML	10 (5.6%)	2 (2.5%)	8 (8.1%)	
SAA	15 (8.3%)	9 (11.1%)	6 (6.1%)	
MDS	15 (8.3%)	8 (9.9%)	7 (7.1%)	
NHL	7 (3.9%)	4 (4.9%)	3 (3.0%)	
Disease status				0.084
First CR	49 (27.2%)	22 (27.2%)	27 (27.3%)	
Beyond first CR	42 (23.3%)	13 (16.0%)	29 (29.3%)	
Others **	89 (49.4%)	46 (56.8%)	43 (43.4%)	
Donor origin				0.014
Match sibling	90 (50.0%)	50 (61.7%)	40 (40.4%)	
Match unrelated	42 (23.3%)	13 (16.0%)	29 (29.3%)	
Mismatched donors ***	48 (26.7%)	18 (22.2%)	30 (30.3%)	
Conditioning regimen				0.515 *
MAC	170 (94.4%)	78 (96.3%)	92 (92.9%)	
RIC	10 (5.6%)	3 (3.7%)	7 (7.1%)	
ATG				0.007
Yes	89 (49.4%)	31 (38.3%)	58 (58.6%)	
No	91 (50.6%)	50 (61.7%)	41 (41.4)	
TBI				0.705
Yes	127 (70.6%)	56 (69.1%)	71 (71.7%)	
No	53 (29.4%)	25 (30.9%)	28 (28.3%)	
Acute GVHD				0.282
Grade 0–1	110 (61.1%)	53 (65.4%)	57 (57.6%)	
Grade 2–4	70 (38.9%)	28 (34.6%)	42 (42.4%)	
CMV serostatus				0.019
R+/D+	139 (77.2%)	64 (79.0%)	75 (75.8%)	
R+/D−	27 (15.0%)	7 (8.6%)	20 (20.2%)	
R−/D+	6 (3.3%)	3 (3.7%)	3 (3.0%)	
R−/D−	8 (4.4%)	7 (8.6%)	1 (1.0%)	

*p*-value was estimated using chi-squared or * Fisher’s exact test. ** others included patient without complete remission and severe aplastic anemia. *** mismatched donors included haploidentical HSCT.

**Table 2:** Incidence rate of CMV reactivation and CMV disease.

	Case Number (%)	Median (days)
CMV reactivation	99 (55.0)	27.58
CMV disease	9 (5.0)	39.56
Pneumonia	3	
Enterocolitis	4	
Retinitis	2	
No CMV reactivation	81 (45.0)	

**Table 3:** Cox proportional hazard regression analysis for CMV reactivation within 100 days after transplantation.

Characteristics	Comparison	Univariate		Multivariate	
		HR (95% CI)	*p*	HR (95% CI)	*p*
Age (years)	≥40 vs. <40	1.06 (0.72, 1.58)	0.765	1.19 (0.79, 1.81)	0.407
Gender	Male vs. female	1.38 (0.93, 2.06)	0.109	1.19 (0.77, 1.82)	0.436
Donor origin	Match unrelated vs. Match sibling	2.04 (1.26, 3.30)	0.004	2.01 (1.24, 3.29)	0.005
	Others vs. Match sibling	1.78 (1.11, 2.87)	0.017	1.69 (1.01, 2.83)	0.045
Conditioning regimen	RIC vs. MAC	1.5 (0.69, 3.23)	0.304	1.8 (0.74, 4.35)	0.194
ATG	Yes vs. No	1.84 (1.23, 2.75)	0.003	-	
TBI	Yes vs. No	1.15 (0.74, 1.78)	0.535	1.23 (0.74, 2.03)	0.429
Acute GVHD	Grade 2–4 vs. Grade 0–1	1.15 (0.77, 1.71)	0.495	1.21 (0.80, 1.84)	0.361
CMV serostatus	Others vs. R+/D+	1.11 (0.70, 1.77)	0.644	1.03 (0.62, 1.71)	0.903

**Table 4:** Characteristics of CMV reactivation and invasive fungal infection (IFI) within 100 days.

Characteristics	Overall (*n* = 180)	No CMV Reactivation (*n* = 81)	CMV Reactivation (*n* = 99)	*p*-Value
Proven IFIs	7	2	5	
Probable IFIs	1	1	0	
Possible IFIs	26	11	15	
Proven, probable and possible IFIs				0.381
Yes	34	14	20	
No	146	67	79	
Early-onset (≤40 days) IFIs				0.237
Yes	20	11	9	
No	160	70	90	
Late-onset (>40 days) IFIs				0.056
Yes	14	3	11	
No	166	78	88	

## Data Availability

The datasets presented in this article are not readily available because of patient confidentiality and participant privacy terms. Requests to access the datasets should be directed to the corresponding author.
